# Associations Between Outcome Resilience and Sociodemographic Factors, Childhood Trauma, Personality Dimensions and Self-Rated Health in Middle-Aged Adults

**DOI:** 10.1007/s12529-022-10061-1

**Published:** 2022-03-04

**Authors:** Patrick Linnemann, Klaus Berger, Henning Teismann

**Affiliations:** Institute of Epidemiology and Social Medicine, University of Münster, Albert-Schweitzer-Campus 1, 48149 Münster, Germany

**Keywords:** Outcome resilience, Stressor, Depressive symptoms, Residualization, Personality dimensions

## Abstract

**Background:**

We examined how sociodemographic factors, childhood trauma, personality dimensions, and self-rated health were associated with outcome resilience and how different stressors influenced depressive symptoms.

**Methods:**

An outcome resilience score for 213 adults was derived by means of a residualization approach. Associations between outcome resilience and sociodemographic and personality factors were evaluated using linear regression. In addition, associations between log-transformed depressive symptoms and the stressors were analyzed using multiple linear regression. A Pearson correlation coefficient between self-rated health and outcome resilience was also computed.

**Results:**

Higher neuroticism was negatively and higher conscientiousness was positively associated with outcome resilience. Better self-rated health was associated with higher outcome resilience. Somatic disease events and onset of chronic mental disorders were associated with more depressive symptoms.

**Conclusions:**

Outcome resilience was significantly related to neuroticism, conscientiousness, and self-rated health. Strong associations between depressive symptoms and the stressors somatic disease event, and chronic mental disorder were observed.

**Supplementary Information:**

The online version contains supplementary material available at 10.1007/s12529-022-10061-1.

## Introduction

Mental health problems (e.g., symptoms of depression, anxiety, or post-traumatic stress disorder (PTSD)) can be influenced and triggered by the experience of stressors such as adverse life events (e.g., deaths of related persons) [1]. The majority of individuals are confronted with traumatic events during their lifetime [2, 3], and about 30% of individuals experience four or more traumatic events during their lifetime [2]. However, not everyone who experiences stressors develops mental problems; most individuals do not develop any [4]. This ability to maintain stable mental health despite stressor experience or to recover fast from a stressor is called (psychological) resilience [5]. Different definitions of resilience have existed and continue to exist, with little uniformity or consensus [6]. One novel claim is that resilience should be viewed less as an individual trait and more as a broader concept influenced at different levels [6]. Resilience, considered as a trait at the personal level, has been actively investigated since the 1980s, and in the beginning, resilience was mainly understood as a relatively stable personality trait (“trait resilience”) [7–9] that can be assessed by self-report questionnaires (e.g., RS-25) [7]. In the last two decades, however, alternative conceptualizations of resilience have emerged; that is, resilience should be regarded as the result of maintaining or regaining mental health while facing stressors. In this context, personality is regarded as one influencing factor among several others (e.g., genetic disposition, socioeconomic status) [1, 10]. This concept is known as “outcome resilience” [1]. If outcome resilience is observed repeatedly over longer periods, it can be considered a process that varies (“process resilience”) [11]. A new concept called “resilience reserve” is based on the understanding of resilience as a multifactorial construct. Resilience is defined as “the sum of physiological processes that protect and compensate for the effect of trauma” [12].

Both the outcome and process resilience approaches have so far neither established nor widely accepted questionnaires to measure resilience [13]. However, other procedures for assessing outcome and process resilience have been developed [11]. The mandatory prerequisite to assess outcome and process resilience is the experience of a stressor [11]. Stressors are physical/social environmental circumstances that challenge a person’s adaptive capabilities and resources [14]. Stressors can be sorted into two different qualitative categories: (i) macro-stressors constitute a significant burden or a potential trauma, like a natural disaster. (ii) Micro-stressors include everyday problems, like problems in relationships [15]. Kalisch and colleagues recently introduced a continuous measure of outcome resilience based on a residualization approach considering the adaptation to macro-and micro-stressors [13].

Among protective factors, it is already known that certain sociodemographic characteristics (e.g., advanced age) are associated with higher probabilities of being outcome-resilient [16]. Another study showed that outcome resilience is associated with a higher level of education [17]. Some studies investigated the relationship between outcome resilience and personality traits employing the Big Five model (openness to experience, conscientiousness, extraversion, agreeableness, neuroticism [18]). The Big Five personality traits model is a highly accepted personality model [19], postulating that five dimensions discriminate most individual personality differences [18]. McDonnell and Semkovska [20] showed that outcome resilience is a mediator between extraversion and neuroticism and depressive symptoms. The authors used a psychometric resilience questionnaire (Connor–Davidson Resilience scale (CD-RISC) [8] and controlled for stressors. The CD-RISC operationalizes resilience as a process, but it can only assess putative protective factors and resources that help maintain or regain mental health despite the experience of stressors [21]. Resilience assessed by the CD-RISC is negatively associated with neuroticism [22, 23].

In contrast, trait resilience was associated with the remaining Big Five dimensions [22]. In addition, Iimura and Taku [24] showed that neuroticism is negatively associated with outcome resilience in adolescents. These authors used the psychometric brief resilience scale (BRS-J) [25] to assess resilience. Compared to the CD-RISC scale, this scale seems more suitable to assess outcome resilience because it considers the individual’s ability to maintain or regain health despite experiencing stressors [21].

Regarding the experience of traumatic events and maltreatment as a child, childhood trauma was found to be negatively associated with trait resilience (RS-11) [26] and that individuals with childhood trauma experience have significantly lower levels of resilience (CD-RISC) in comparison with healthy controls [27]. Most of the studies discussed focused on the experience of specific stressors. However, recording a wider range of potential stressors over a certain period appears reasonable because it seems unlikely that one stressor is not followed by other stressors [1, 13].

It is well-known that the experience of stressors can affect mental health [28]. However, how different individuals react to specific stressors is very diverse, and it is difficult to predict how an individual will react to a specific stressor [13]. Detailed findings on the effects of different trauma types and the mental health outcome PTSD already exist. For example, sexual violence in a partnership is associated with a high risk of PTSD, and the death of a loved one is associated with a low risk of PTSD [3]. Some studies took a more differentiated look at how specific stressors are dealt with and how these stressors are evaluated qualitatively. For instance, for adults, the death of a parent is associated with poor mental health status [29]. Middleton and colleagues [30] found that adults perceive the handling of a child’s bereavement as most intense, followed by a spouse’s bereavement. In contrast, the least intense mourning process occurs when adults mourn over their parents. Separation from a partner is associated with an increased risk of poorer health, including increased mortality [31]. Nevertheless, overall, most persons cope well with separations [31].

Significant somatic disease events may influence mental health [32]. However, the impact of experiencing a severe somatic disease can also have very different effects on an affected person’s depressive symptoms. For example, the change in depressive symptoms after experiencing a myocardial infarction is very heterogeneous, with the most common adjustment being resilience [33]. Note, however, that studies revealed a bidirectional relationship between somatic chronic diseases and mental health problems [34, 35]. However, little is known about the underlying mechanisms linking chronic somatic diseases and mental health problems; thus, further research is needed [36, 37].

Here, we consider different somatic disorders (e.g., heart attack, stroke), including first onset of chronic diseases (e.g., diabetes, cardiac insufficiency, pulmonary disease) as well as chronic mental disorders (e.g., addiction). One study [38] examined the impact of cancer, stroke, heart disease, and lung disease on mental health. The study also distinguished whether individuals had experienced only one or a higher number of significant disease events. The study showed that experiencing significant disease events led to a reduction in mental health and increased mental health problems regardless of the number of events. The number of stressors did not influence resilience. Moreover, meta-analyses showed that chronic disease is associated with decreased resilience, measured by different resilience questionnaires, and conceptualized mostly as trait resilience [39, 40].

Another interesting topic is the association between resilience and self-rated health, observed in diverse settings. For patients recovering from arthroplasty, a positive correlation between resilience (CD-RISC) [8] and self-rated health (EQ-VAS) [41] was found [42]. A similar result was seen for HIV-positive South Africans [43]. Losoi and colleagues [44] found a significant correlation between self-rated health and resilience only in women. These studies have in common that resilience was measured with a questionnaire and understood as a trait. Further studies investigated the link between self-rated health and mental health for persons who have experienced severe life events. Burns and colleagues [45] found that psychological well-being components (including resilience) were significant predictors of subjective well-being and that resilience is negatively associated with depression and anxiety [45]. Cosco and colleagues [46] showed that experiencing more psychosocial challenges is associated with more mental challenges in advanced adulthood.

Based on a sample of community-dwelling adults, the present study aimed to investigate (i) how certain factors are associated with outcome resilience; (ii) how different categories of stressors influence mental health problems, expressed as depressive symptoms; and (iii) how self-rated health is associated with outcome resilience. In consideration of previous findings, we expect that (iv) higher values on the Big Five dimension neuroticism and having lived through childhood trauma would negatively influence outcome resilience, whereas older age, higher educational attainment, and higher values on other Big Five dimensions would have a positive influence. Moreover, we expect that (v) sex would influence outcome resilience in one or the other direction and that (vi) all the stressors would exacerbate mental health problems, expressed as depressive symptoms. Finally, (vii) we expect to find a relationship between outcome resilience and self-reported health.

## Methods

### Study Population

The BiDirect study [47, 48] is a large, observational, longitudinal study originally designed to investigate the bidirectional relationship between depression and (subclinical) arteriosclerosis. The study was conducted from 2010 to 2020 and incorporated a baseline and three follow-up examinations. BiDirect integrates three cohorts of adults aged 35–65 years at recruitment: (i) patients hospitalized with an acute episode of depression during recruitment, (ii) patients who experienced an acute coronary event 3 to 4 months before recruitment, and (iii) community-dwelling control subjects randomly invited from the registry of the city of Münster, Germany. Participants in this analysis are members of the control cohort (i) who took part in the first BiDirect follow-up examination (*N* = 800) and (ii) who had experienced at least one macro-stressor (*N* = 213) in the period between baseline (July 2010 to June 2013) and first follow-up examinations (July 2013 to December 2015). Participants with missing values in the respective outcomes of interest or in predictors were excluded. Ethical approval was obtained by the ethics committee of the University of Münster and the Westphalian Chamber of Physicians in Münster, Germany. All participants gave their informed consent in written form.

### Outcome

An outcome-resilient individual was defined as a person reporting less depressive symptoms than predicted by a linear model based on the number of stressors experienced. Likewise, a non-outcome-resilient individual was defined as a person reporting more depressive symptoms. Prior stressor experience is a prerequisite for constructing an outcome resilience measure.

### Stressor Count

In the present study, we collected psychosocial and disease-related stressors between the BiDirect baseline and the first BiDirect follow-up examination, thus incident events over a period of 2.7 years on average. Data on psychosocial and disease-related stressors were self-reported by participants. For each disease-related stressor, a physician diagnosis of the respective disease in the period since the baseline examination was assessed, its frequency and its status as prevalent or incident determined. We grouped the psychosocial stressors into the following qualitative categories: (1) separations from partners/divorces; (2) deaths of partners/spouses/children; and (3) deaths of parents. In addition, we included disease-related stressors, grouped into the following categories: (1) first onset of chronic somatic disease (including diagnoses of diabetes, kidney disease, lung disease, Parkinson’s disease, and addiction disease); (2) first onset of chronic mental disorders (including depression, anxiety disorder, and addiction disease); and (3) occurrence of somatic disease events (including diagnoses of myocardial infarction, stroke, cancer, and thrombosis). All information about the experienced stressors and diseases were provided by participant self-reports.

### Depressive Symptoms

We adopted the perspective that, based on theory of positive clinical psychology and previous findings [49], depressive symptoms form one pole and well-being the other of a continuum of mental health. To measure depressive symptoms after experiencing stressors, we used the Center for Epidemiologic Studies Depression Scale (CES-D) [50]. The CES-D score has a good one-dimensional fit [51] and is suitable, with certain restrictions, for measuring a continuum ranging from well-being to depression [49, 52]. The CES-D consists of 20 items that refer to the past week. Answers to the items can be given on a four-point rating scale (0 = “Rarely or none of the time (less than one day)” through 3 = “Most or all of the time (5–7 days)”).

### Self-Reported Health

Here, the perceived health status was assessed by the EQ-5D-3L [41] visual analog scale (EQ-VAS), ranging from zero (“the worst health you can imagine”) to hundred (“the best health you can imagine”).

### Assessment of Outcome Resilience Employing SR Score

To operationalize outcome resilience as a continuous measure, we derived an SR score as suggested by Kalisch and colleagues [13]. The SR score is based on the assumption that there is a positive linear relationship between the number of stressors experienced over a certain period and the extent of mental health problems measured at the end of the period. After regressing the mental health measure (the outcome, here, the CES-D score) onto the number of stressors experienced (the predictor), the regression line would represent the “norm” of dealing with a specific number of stressors. The regression residuals form the SR score. Here, a positive residual (or likewise, a positive SR score) means that a person is *less* resilient than the norm (because the CES-D score was higher or worse than predicted by the model). In contrast to Kalisch and colleagues [13], we omitted micro-stressors but focused on macro-stressors.

### Covariates

Sociodemographic covariates of interest included age, sex, and the number of years in full-time education. Moreover, we included self-reported childhood trauma (assessed via the CTS scale) [53] and the Big Five personality dimensions [18] (assessed via the BFI-S scale) [54]. The Big Five Inventory short (BFI-S) measures the five central personality dimensions (neuroticism, extraversion, openness to experience, agreeableness, conscientiousness [18]) using 15 items.

Demographic and stressor characteristics and their descriptive statistics are displayed in Table 1. The majority of participants were middle-aged (56.34 ± 7.70), the sex ratio was well-balanced (48.4% female), and participants had high average education years (14.64 ± 2.78). The most frequently experienced stressor category was a parent’s death (106), followed by the onset of chronic somatic disease (40) and partner/spouse separation (38).

**Table 1:** Characteristics and scores

*N*	213
Age in years^1^	56.34 ± 7.70
Sex = female^2^	103 (48.4)
Years in full-time education	14.64 ± 2.78
Time in study (years)	2.72 ± 0.37
CES-D^3^ score	10.87 ± 8.25
EQ5D-3L-VAS^4^ score	75.67 ± 16.74
CTS^5^ score	7.93 ± 2.93
BFI-S^6^ neuroticism score	10.64 ± 4.35
BFI-S extraversion score	13.97 ± 3.88
BFI-S conscientiousness score	17.05 ± 3.01
BFI-S openness score	14.05 ± 3.93
BFI-S agreeableness score	16.58 ± 2.82
Amount of separations	
0	175 (82.2)
1	34 (16.0)
2	4 (1.9)
Amount of deaths spouse/ child	
0	203 (95.3)
1	10 (4.9)
Amount of deaths parents	
0	107 (50.2)
1	100 (46.9)
2	6 (2.8)
Amount of somatic disease events	
0	188 (86.4)
1	27 (12.7)
2	2 (0.9)
Amount of chronic mental disorders	
0	184 (86.4)
1	27 (12.7)
2	2 (0.9)
Amount of chronic somatic diseases	
0	173 (81.2)
1	38 (17.8)
2	2 (0.9)

^1^Continuous variables are described by mean (± SD)

^2^Categorical variables are described by *N* (%)

^3^Center for Epidemiologic Studies Depression Scale

^4^EQ5D-3L visual analog scale

^5^Childhood Trauma Screener

^6^Big Five Inventory Short

### Data Analysis

We analyzed the data with R [55] using R Studio [56] following the steps recommended by Fife [57].

First, to evaluate whether the numbers of stressors from different categories influenced depressive symptoms (CES-D score), we conducted a multiple linear regression analysis. In order to account for the non-normally distributed, positively skewed dependent variable (CES-D score) (Supplementary material Fig. 1), we log-transformed the CES-D score, aiming for approximate normality. Independent variables were the numbers of stressors (continuous) from six categories experienced between the BiDirect baseline and first follow-up examinations and the time interval (continuous) between the baseline and first follow-up examinations. Noteworthy, this time interval varied between individuals (range, 2.1 years to 4.5 years; mean, 2.7 years; SD, 0.4 years) due to the composition of BiDirect. In addition, we compared our subsample with the remaining sample to reveal a potential selection bias. The results showed no significant differences except the expected effect of depressive symptoms (CES-D sum score, *p* < 0.001). Thus, further analyses were conducted.

**Fig. 1:**
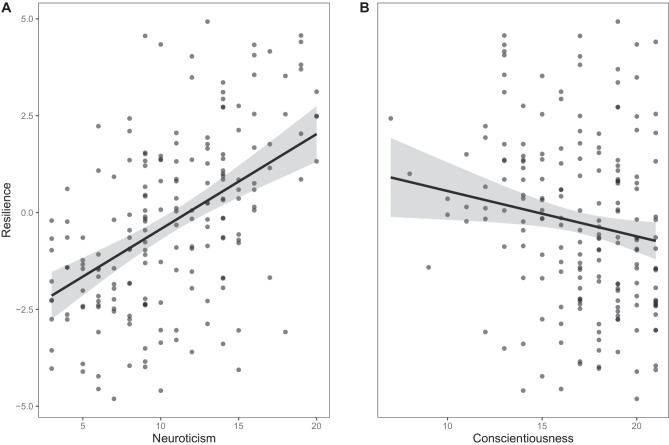
Associations of neuroticism and conscientiousness with outcome resilience (SR score), in each case adjusted for the other predictors in the model. A positive resilience score means here that a person is less resilient and vice versa. Points denote partial residuals. Gray lines denote linear trends. Gray shades denote 95% confidence intervals of the linear trends

Second, we analyzed the relationships between outcome resilience and covariates of interest using multiple linear regression analysis. The dependent variable was outcome resilience operationalized as the SR score, representing the quasi-Poisson regression residuals computed in step one (residualization approach). Independent variables were age (continuous), sex (categorical), number of years in full-time education, childhood trauma, neuroticism, extraversion, openness, agreeableness, and conscientiousness (in each case continuous).

Third, we computed a Pearson correlation coefficient to determine the association between outcome resilience (SR score) and self-reported health (EQ-VAS).

## Results

Two hundred thirteen individuals from the BiDirect control cohort were eligible for the analyses. The results of the multiple linear regression analysis computed to predict log-transformed depressive symptoms (CES-D score) from the numbers of stressors from different categories and participation time are depicted in Table 2. The model became significant (*F*(9, 191) = 5.37, *p* < 0.001, with an *R*^2^ of 0.16, Cohen’s *f*^2^ of 0.19)). There were significant effects of somatic disease events (estimate = 0.38, 95% CI = 0.03, 0.73), and onset of chronic mental disorders (estimate = 0.74, 95% CI = 0.43, 1.05). Because the dependent variable was log-transformed, for instance, the increase of one point in somatic disease events means that the log-transformed CES-D increased by 0.38 points; exponentiating this leads to an increase of 1.46 CES-D points. We used an alpha value of *p* < 0.05 and therefore a confidence level of 0.95. The remaining predictors did not reach statistical significance. Higher numbers of stressors were associated with more depressive symptoms (higher CES-D score).

**Table 2:** Results of the multiple linear regression analysis with log-transformed depressive symptoms (CES-D score) as outcome

*Predictors*	*Beta coefficients*	*std. beta coefficients*	*95% CI*	*p*
Intercept	2.64	0.00	1.83, 3.45	** < 0.001**
Time in study (years)	− 0.25	− 0.11	− 0.56, 0.05	0.101
Separations	0.26	0.14	− 0.02, 0.55	0.067
Death spouse/child	0.37	0.10	− 0.16, 0.90	0.166
Death parents	− 0.02	− 0.01	− 0.27, 0.24	0.900
Somatic disease events	0.38	0.16	0.03, 0.73	**0.035**
Chronic mental disorders	0.74	0.33	0.43, 1.05	** < 0.001**
Chronic somatic diseases	0.16	0.08	− 0.12, 0.44	0.262
Observations	199			
*R*^2^	0.16 (*p* < **0.001**)			

The results of the multiple linear regression analysis with the SR score as outcome and sex, age, education, childhood trauma, and the personality dimensions as predictors are shown in Table 3. The model became significant (*F*(9, 179) = 7.86, *p* < 0.001, with an *R*^2^ of 0.28, Cohen’s *f*^2^ of 0.39)). There were significant effects of neuroticism (estimate = 0.07, 95% CI = 0.04, 0.10) and conscientiousness (estimate =  − 0.04, 95% CI =  − 0.08, − 0.01). We used an alpha value of *p* < 0.05 and therefore a confidence level of 0.95. The remaining predictors did not reach significance. While higher neuroticism was associated with lower outcome resilience, higher conscientiousness was associated with higher outcome resilience. Plots of the associations of neuroticism and conscientiousness with the SR score, adjusted for the other predictors in the model, are given in Fig. 1. Figure 1 depicts that higher levels of neuroticism lead to a higher SR score or lower outcome resilience, respectively. Furthermore, the figure shows that higher levels of conscientiousness lead to a lower SR score and thus higher outcome resilience.

**Table 3:** Results of the multiple linear regression analysis with outcome resilience (SR score) as outcome

*Predictors*	*Beta coefficients*	*std. beta coefficients*	*95% CI*	*p*
Intercept	− 0.00	0.00	− 1.32, 1.31	0.996
Age (in years)	0.05	0.03	− 0.16, 0.26	0.638
Sex (female)	0.01	0.05	− 0.01, 0.02	0.443
Years in full-time education	− 0.01	− 0.05	− 0.05, 0.02	0.502
CTS score	0.03	0.10	− 0.01, 0.06	0.119
BFI-S neuroticism score	0.07	0.40	0.04, 0.10	** < 0.001**
BFI-S extraversion score	− 0.01	− 0.07	− 0.04, 0.02	0.372
BFI-S conscientiousness score	− 0.04	− 0.16	− 0.08, − 0.01	**0.025**
BFI-S openness score	0.00	0.01	− 0.03, 0.03	0.894
BFI-S agreeableness score	− 0.02	− 0.07	− 0.06, 0.02	0.346
Observations	189			
*R*^2^	0.28 (*p* < **0.001**)			

The Pearson correlation coefficient between outcome resilience (SR score) and self-rated health (EQ-VAS) was − 0.49 (*p* < 0.001); thus, better self-rated health was associated with higher outcome resilience (i.e., a lower SR score) and vice versa. The supplementary materials also show the correlation between outcome resilience and self-rated health status (Supplementary material Fig. 2).

## Discussion

In a sample drawn from a population-based cohort, we examined if and how specific resilience factors influenced outcome resilience. Second, we investigated the influence of defined macro-stressors on depressive symptoms. And third, we explored the relationship between self-rated health and outcome resilience.

Concerning outcome resilience, we hypothesized that higher values on both neuroticism and having lived through childhood trauma would negatively influence outcome resilience, while older age, a higher number of years in full-time education, and higher values on the remaining personality dimensions would positively affect outcome resilience. In addition, we expected an influence of sex. Although we found no differences of sex regarding outcome resilience and adaptation toward stressors, several previous studies found sex differences in coping with specific stressors [58–60]. The present study showed that higher neuroticism was negatively associated with outcome resilience, whereas higher conscientiousness had a positive influence. The present finding of a relationship between neuroticism and outcome resilience aligns with previous findings. Neuroticism was negatively associated with three different conceptualizations (outcome, process, and trait) of resilience [24, 61, 62]. From a theoretical perspective, neuroticism is widely regarded as being associated with an inadequate response to stress [63]. Moreover, higher levels of neuroticism are associated with a higher amount of mental disorders [64]. The relationship between conscientiousness and trait resilience is well-established [62]. The personality dimension conscientiousness describes differences in individuals regarding their expressions and tendencies toward responsibility, diligence, neatness, self-control, and conformity to rules [65]. Contrary to neuroticism, conscientiousness is associated with abilities and traits that are considered to promote resilience [22], such as the ability to cope well with stressors [66] or a high level of self-efficacy [67]. Individuals who show high levels of self-efficacy have the conviction in their ability to control their motivation, behavior, and social environment [68] and tend to actively pursue solutions to deal with stress [22]. A related concept to self-efficacy is the concept of internal locus of control [69], which contains the belief of having the control over one’s life. Previous research has revealed that persons with a strong internal locus of control possess the ability to deal with numerous extremely stressful life events [70]. An association between internal locus of control and resilience was already found [71]. However, it should be kept in mind that only a fraction of possible influencing factors conducive to coping with the experience of stressors were collected and analyzed in the current study. Successful and thus resilient coping with stressors and adverse events is widely considered an interplay at different levels (e.g., biological, environmental factors) [11, 72]. For example, the expression of a person’s resilience could depend strongly on the level of perceived social support, connectedness, and loneliness, which was not investigated here. It has already been shown that more loneliness is associated with lower resilience. This has been explained by the fact that resilient individuals have intrapersonal and interpersonal resources that facilitate coping with loneliness [73]. It has also been shown that lower levels of loneliness and higher levels of social support are associated with more resilience [74]. Social support and resilience share psychological and behavioral mechanisms that should help deal with bad events. They should promote, for example, that adverse events are perceived as less threatening and that persons perceive an increased sense of control [75]. Another aspect under which the current results should be considered is the aspect of heterogeneous and inconsistent definition and conceptualization of resilience [6, 76] and the concomitant difficulty of comparing results. Nevertheless, our findings contributed to understanding the relationship between resilience, as the positive adaptation after experiencing adverse events, and neuroticism and conscientiousness as personality dimensions. Furthermore, these findings fit well with previous literature, regardless of how resilience is conceptualized.

About the influence of macro-stressors on depressive symptoms, we hypothesized that all stressors considered in the present study would intensify depressive symptoms. The findings revealed that the first onset of chronic mental disorder, and the experience of a somatic disease event indeed exacerbated depressive symptoms. The null hypothesis that the remaining stressors did not influence depressive symptoms could not be rejected. The finding that there was insufficient evidence for some stressors could be due to the small sample size. Therefore, further studies with larger subsamples are necessary to obtain the necessary statistical power to analyze specific stressors such as the loss of a child or partner.

The first finding, indicating that both the first onset of a chronic mental disorder and the experience of a somatic disease event intensified depressive symptoms, is not surprising, since the reciprocal link between somatic and mental health is already well-established [86, 87]. Past somatic health has strong direct and indirect impacts on the present mental health and vice versa [32]. Here, we distinguished three categories of diseases. The first two categories consisted of rather chronic diseases (diabetes, kidney disease, lung disease, anorexia, Parkinson's disease, MDD, addiction disease, and anxiety disorder), and the third category consisted of somatic disease events (myocardial infarction, stroke, cancer, and thrombosis). The categories of mental disorders and somatic disease events intensified depressive symptoms confirm earlier findings on the influence of certain diseases on mental health problems. For chronic mental disorders, it has already been reported that major depressive disorder [50], anorexia [77], Parkinson’s disease [78], anxiety disease [79], and addiction disease [80] are associated with poorer mental health and more mental health problems. Likewise, it was already reported that somatic disease events intensified mental health problems (myocardial infarction [81], stroke [82], cancer [83], and thrombosis [84]). In terms of practical implications, the present results indicated that the onset of chronic mental disorder led to an increase of 2.1 CES-D points, while the experience of a somatic disease event led to an increase of 1.46 CES-D points. In our view, consistent with our statements above, the influences of the respective stressor categories on the CES-D score are neglectable and not clinically relevant. From our point of view, the present findings on the impact of diseases (chronic mental disorder or eventful) on depressive symptoms complement the existing literature. In the future, detailed information about the effects of different stressful events on mental health should be studied further, as is already the case for the effects of trauma types on PTSD [3]. Moreover, the findings indicate that grouping stressors such as diseases into qualitative categories are worthwhile when constructing an SR score via a residualization approach. In line with former studies [43, 44], the present study detected that better outcome resilience is also associated with better self-rated health and vice versa in individuals derived from a population-based cohort.

### Strengths and Limitations

The present study operationalized and conceptualized resilience in an up-to-date manner, employing the outcome resilience approach [1]. Outcome resilience was assessed by a metric measure, the stressor reactivity score, which is theoretically based on the residualization approach [13]. The SR score was composed of a wider range of macro-stressors experienced over around 3 years. The current study examined only a subsample of individuals who experienced a stressor in a designated period of time from a population-based cohort. Nevertheless, we assume that our results are generalizable to residents of a major Western European city, as we assume that all participants in the control cohort had an equal probability of having experienced a stressor during the designated period.

Despite our efforts to include a broad spectrum of relevant macro-stressors to compute the SR score, we cannot rule out that significant stressors have remained unnoticed. For instance, the present study neglected work-related macro-stressors like job loss or housing related macro-stressors like area-level deprivation. Further, we did not consider any micro-stressors or daily hassles, which could significantly moderate mental health outcomes. Due to a relatively small sample size in the present study, some stressors were experienced less frequently and were predicted with little precision. In addition, it is important to consider that individuals who have experienced a particular stressor or illness may be at increased risk of experiencing stressors or illnesses again compared to individuals without these experiences. Moreover, the qualitative sorting of stressors into categories may also be considered critical. As usual for longitudinal observational studies, there was some loss of participants from the first to the second survey time point. These losses to follow-up could have biased the selected population in the present study. It is possible, for example, that people who have experienced particularly stressful life events have not participated in the study. Besides, the mental disorders assessed in the present study were limited exclusively to depressive symptoms.

## Conclusions

The present study found a negative influence of higher neuroticism and a positive influence of higher conscientiousness on outcome resilience. The finding regarding neuroticism is consistent with previous findings. The finding regarding conscientiousness contributes to novel insight into the structure of outcome resilience. Also, in line with previous findings, we found that chronic mental disorders and somatic disease events were associated with increased depressive symptoms. Lastly, we found that outcome resilience had a positive relationship with self-rated health. Since some of our findings overlap with results from the trait resilience approach, we think it would be helpful to investigate the relationship between these two constructs more intensively in future studies. Our results also imply that health practitioners should pay extra attention to individuals with recent diseases events, chronic mental disorders, and to individuals high in neuroticism, since these individuals appear somewhat less resilient and therefore may have or are about to develop relevant mental health problems. In particular, individuals with high levels of neuroticism could benefit preventively from offers to strengthen resilience, since neuroticism levels are quite stable over time [85] and thus easily and time independently detectable.

## Supplementary Information

Below is the link to the electronic supplementary material.

Supplementary file1 (DOCX 450 KB)
