# Supplementary material for: Associations Between Outcome Resilience and Sociodemographic Factors, Childhood Trauma, Personality Dimensions and Self-Rated Health in Middle-Aged Adults
Source: Int J Behav Med. 2022 Mar 4;29(6):796–806. doi: 10.1007/s12529-022-10061-1 (PMC9684253; doi:10.1007/s12529-022-10061-1)
Supplement: Supplementary file 1 — Supplementary file1 (DOCX 450 KB) [file 12529_2022_10061_MOESM1_ESM.docx]

**Supplemental Materials**

**Supplemental Figure 1**

**Fig 1.** Distribution of the CES-D score


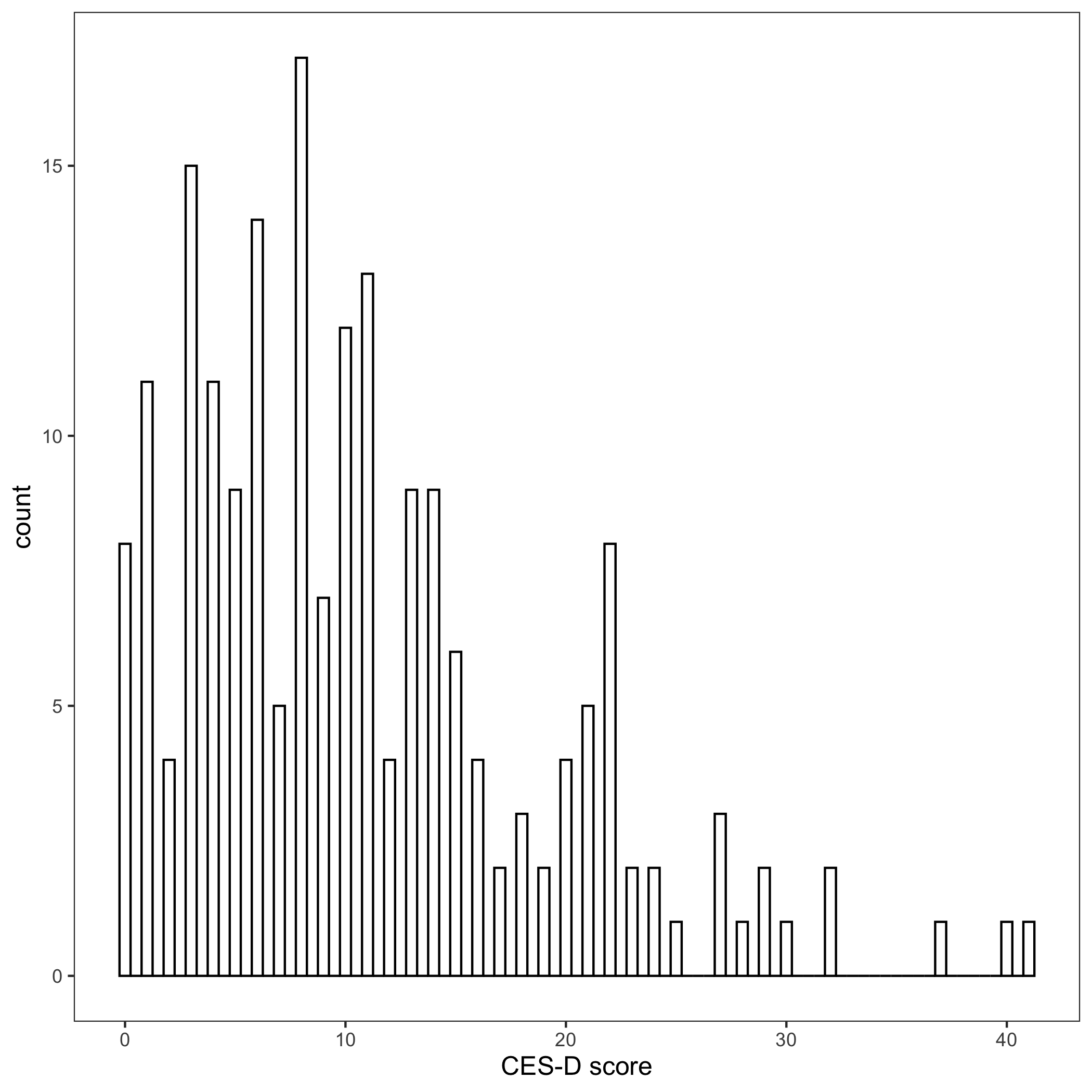


**Supplemental Figure 2**


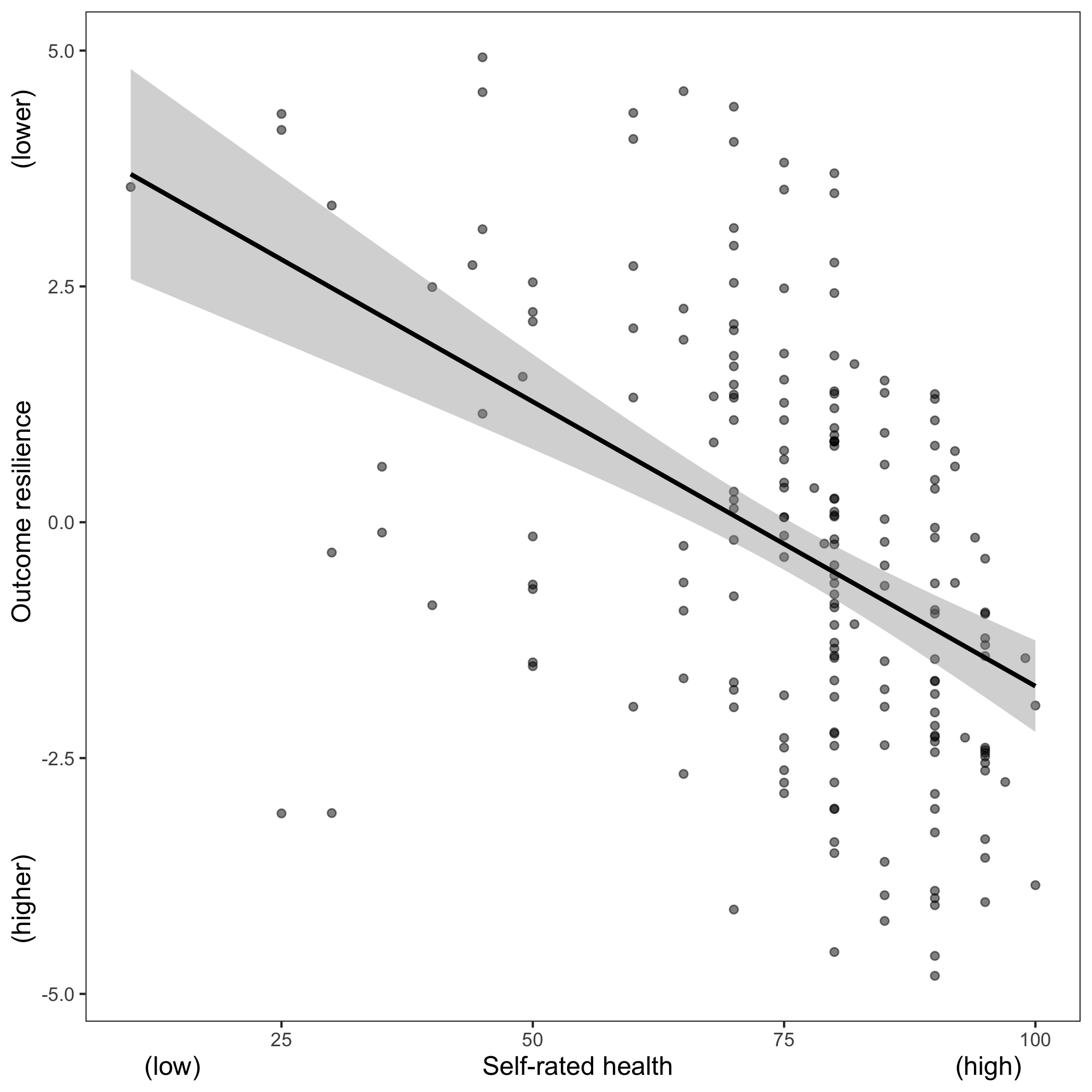


**Fig 2.** Association between outcome resilience (SR score) and self-rated health (EQ5D-VAS). Negative values denote higher outcome resilience, positive values denote lower outcome resilience. Points denote individual data points. The black line represents the linear relationship between outcome resilience and self-rated health. The gray shaded area denotes the 95% confidence interval of the linear trend.
